# Hydration and Biodistribution of Zwitterionic Dendrimers Conjugating a Sulfobetaine Monomer and Polymers

**DOI:** 10.1021/acs.langmuir.4c04276

**Published:** 2025-01-08

**Authors:** Chie Kojima, Rikuto Hirata, Nanako Dei, Hao He, Yuka Ikemoto, Akikazu Matsumoto

**Affiliations:** † Department of Applied Chemistry, Graduate School of Engineering, Osaka Metropolitan University, 1-1 Gakuen-cho, Naka-ku, Sakai, Osaka 599-8531, Japan; ‡ Department of Materials Science and Engineering, School of Materials and Chemical Technology, Institute of Science Tokyo, 4259 Nagatsuta-cho, Midori-ku, Yokohama, Kanagawa 226-8503, Japan; § Spectroscopy Division, Japan Synchrotron Radiation Research Institute, 1-1-1 Kouto, Sayo-cho, Sayo-gun, Hyogo 679-5198, Japan

## Abstract

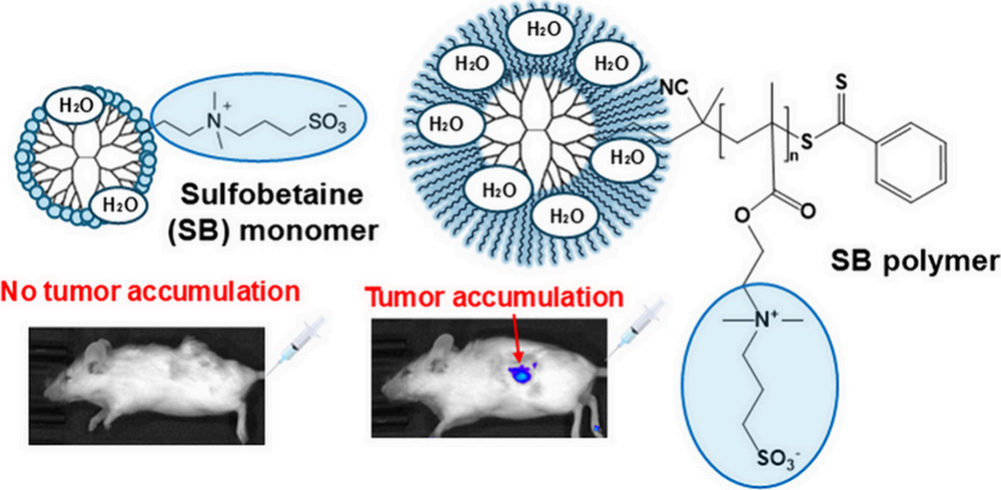

Zwitterionic polymers exhibit strong hydration, high biocompatibility, and antifouling properties. Dendrimers are regularly branched polymers, which are used in the drug delivery system (DDS). In this study, we synthesized zwitterionic monomer- and polymer-conjugated dendrimers as a biocompatible nanoparticle to investigate the relation between the hydration property and biodistribution. A sulfobetaine monomer (SBM) was conjugated at the termini of the polyamidoamine (PAMAM) dendrimer. Polysulfobetaines (PSBs) were produced by reversible addition–fragmentation chain transfer polymerization and were also conjugated at the termini. Intermediate water, that is, water molecules loosely bound to the material, can be estimated from the melting peaks at less than 0 °C in differential scanning calorimetry (DSC) measurement. Our DSC results showed that the PSB-conjugated dendrimers (PSM-dens) contained more intermediate water than the SBM-conjugated dendrimer (SBM-den). PSB-dens accumulated in the tumor after intravenous administration, but SBM-den did not. These suggested that the amount of intermediate water, that is, the hydration property, was related to the biodistribution of the zwitterionic dendrimers. This relation is a possible design criterion for drug carriers. PSB-dens accumulated in the tumor even after the second injection, possibly overcoming the accelerated blood clearance observed with poly(ethylene glycol)-modified nanoparticles. Thus, this kind of zwitterionic polymer-conjugated dendrimer is useful for the DDS in cancer treatment.

## Introduction

Drug delivery systems (DDSs) are technologies that deliver therapeutic agents to the desired site of action precisely and effectively through which drug action and side effects can be enhanced and reduced, respectively. DDSs are important in cancer therapy, and drug carriers with prolonged blood circulation can accumulate in tumors via the enhanced permeability and retention (EPR) effect.^1^ Several types of poly(ethylene glycol) (PEG)-modified (PEGylated) nanoparticles have been developed as drug carriers for cancer chemotherapy because PEG exhibits antifouling effects. Some PEGylated nanoparticles, such as PEGylated liposomes, have been used clinically.^2,3^ However, PEGylated liposomes exhibit an accelerated blood clearance (ABC) phenomenon in which PEGylated nanoparticles are removed from the blood after multiple injections because of the production of anti-PEG antibodies after the first injection and subsequent anti-PEG immunological reactions after multiple administrations. Overcoming the ABC phenomenon is indispensable in a DDS.^2−6^

Zwitterionic polymers, which contain both positively and negatively charged groups, have also been developed as biocompatible polymers for use in various biomedical applications, including DDS.^2,7−10^ Phospholipid-mimetic polymers, synthesized using 2-methacryloyloxyethyl phosphorylcholine (MPC), were developed by Ishihara et al.^11^ MPC polymers contain phosphobetaine (PB) groups that exhibit antifouling properties. Polymers containing sulfobetaine (SB) and carboxybetaine (CB) have been studied as biocompatible polymers too.^2,7−10^ It has been reported that the strong hydration of zwitterionic polymers leads to their antifouling properties.^2,7−10,12,13^ Hydrated water molecules are classified into three types in an intermediate water concept: non-freezing water, free water, and intermediate water.^13−15^ From differential scanning calorimetry (DSC) analysis, non-freezing water, which interacts strongly with materials, does not freeze even at −100 °C. Free water, which interacts scarcely with materials, melts at 0 °C. Additionally, the melting peak below 0 °C is observed, which corresponds to intermediate water. Some reports indicate that intermediate-water-rich polymers exhibit excellent blood compatibility, and some coating materials for medical devices, such as poly(2-methoxyethyl acrylate), have been developed on the basis of the intermediate water concept.^13−15^

Because zwitterionic polymers can be used as alternatives to PEG, these polymers have been modified on the surface of nanoparticles to produce drug carriers with prolonged blood circulation properties.^3^ Many types of nanoparticles, such as liposomes and micelles, are used as drug carriers. Moreover, dendrimers, regularly branched polymers with well-defined structures, can be used as drug carriers. In contrast to liposomes and micelles, dendrimers are unimolecular nanoparticles whose size and terminal number can be controlled by dendrimer generation.^16−19^ Zwitterionic compounds with CB, SB, and PB have been modified at the termini of dendrimers to add biocompatibility to the dendrimers.^20−22^ Some zwitterionic dendrimers conjugating CB and SB showed prolonged blood circulation and accumulation in tumors and could function as drug carriers for cancer therapy and as imaging probes for cancer diagnosis.^22−26^ However, to the best of our knowledge, there have been no reports on the hydration behavior of zwitterionic dendrimers and a comparison of zwitterionic monomer- and polymer-conjugated dendrimers in biodistribution.

**Figure 1:**
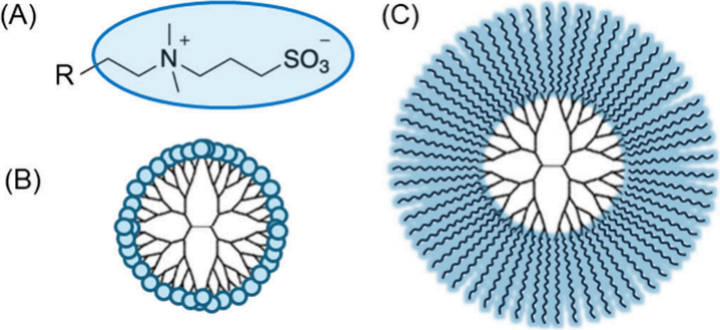
Structures of (A) SB and zwitterionic dendrimers conjugating (B) SBM and (C) PSB.

In this study, we investigated the relation between the hydration and biodistribution of zwitterionic dendrimers. The sulfobetaine monomer (SBM)- and polysulfobetaine (PSB)-conjugated dendrimers (SBM-den and PSB-den) were designed and synthesized (Figure 1). The hydration state was analyzed by DSC measurements of the hydration samples to estimate the amount of intermediate water. The Fourier transform infrared spectroscopy (FTIR) measurements of these hydrated dendrimers were also performed to examine their hydration behaviors, and the cytotoxicity of these dendrimers was examined. In addition, these dendrimers were labeled with indocyanine green (ICG) and intravenously injected into tumor-bearing mice. Biodistribution, particularly tumor accumulation, was examined using *in vivo* and *ex vivo* fluorescence imaging. The same experiment was also conducted after the second injection to examine whether the zwitterionic dendrimers exhibited the ABC phenomenon.

## Experimental Section

### Synthesis of SBM- and PSB-Conjugated Dendrimers

3-[(3-Acrylamidopropyl)dimethylammonio]propane-1-sulfonate (DMAAPS) was synthesized according to the previous report.^27^ The amino-terminal polyamidoamine (PAMAM) G5 dendrimer (Sigma-Aldrich Co. LLC, St. Louis, MO, U.S.A.) was reacted with 180 or 122 equiv of DMAAPS in aqueous NaCl solution at 70 °C for 48 h. After dialysis, the SBM-dens were obtained in yields of 5–70%.

3-[[2-(Methacryloyloxy)ethyl]dimethylammonio]propane-1-sulfonate (DMAPS, 360 mg, 1.29 mmol, Tokyo Chemical Industry Co. Ltd., Tokyo, Japan), 4,4′-azobis(4-cyanovaleric acid) (ACVA, 30 mg, 0.10 mmol), and 4-cyano-4-[(phenylcarbonothioyl)thio]pentanoic acid (CTP, 36 mg, 0.13 mmol) were dissolved in 3 mL of methanol. The freezing–degassing–thawing cycle was repeated 4 times. Polymerization was then performed at 60 °C for 20 h. After evaporation, the crude polymer was dissolved in water and purified via reprecipitation using ethanol. PSB was collected by centrifugation and freeze-dried. Yields of 81 and 93% were obtained with PSB4.5k and PSB5.0k, respectively. The amino-terminal PAMAM G4 dendrimer (Sigma-Aldrich) was reacted with 64 equiv of PSB4.5k or PSB5.0k using 192 equiv of 4-(4,6-dimethoxy-1,3,5-triazin-2-yl)-4-methylmorpholinium chloride (DMT-MM) in 3 mL of phosphate-buffered saline (PBS) at room temperature for 72 h. The dendrimers were purified by ultrafiltration (molecular weight cutoff of 30 000) in PBS and pure water. The PSB-dens were obtained by freeze-drying. Yields of 41 and 49% were obtained for PSB4.5k53-den and PSB5.0k30-den, respectively.

### Characterization

The synthesized compounds were characterized by ^1^H nuclear magnetic resonance (NMR) spectroscopy using an ECX-400 instrument (JEOL, Ltd., Tokyo, Japan) or an Ascend 400 Nanobay spectrometer (Bruker, Billerica, MA, U.S.A.).

The synthesized polymers and dendrimers were characterized using gel permeation chromatography (GPC). Polymers flowed into tandemly linked TSK gel GMPW_XL_ and TSK gel α-3000 columns (Tosoh Corporation, Tokyo, Japan) at 25 °C at a flow rate of 0.5 mL/min, eluting the mixture of 0.1 M (NH_4_)_2_SO_4_ aqueous solution and acetonitrile at the volume ratio of 4:1. The polymers were detected at 220 nm using an ultraviolet (UV) detector (JASCO Corporation, Japan).

The fluorescamine assay was performed, as described in our previous investigation.^28^ Briefly, 0.3 mg/mL of a fluorescamine solution (200 μL, acetone) was added to 0.4 mg/mL of a dendrimer solution (4 mL, 0.5 M borate buffer at pH 8–9). After vortexing, the fluorescence intensity of the solution was measured at 480 nm with excitation at 390 nm using a FP-6200 spectrofluorometer (JASCO). The number of remaining amino group in PSB-dens was estimated from the standard curve of the dendrimer (Figure S1 of the Supporting Information).

### DSC Measurements

DSC analysis was performed using DSC250 RCS90 (TA Instruments-Waters LLC, New Castle, DE, U.S.A.) or DSC-60 Plus (Shimadzu Corporation, Kyoto, Japan), as described in our previous report.^29^ Hydrated polymer samples were prepared by dissolving the dried polymers in pure water for more than 1 day. Approximately 3–5 mg of the hydrated samples was placed in an aluminum container and sealed after adjusting the water content (WC) by air drying for several hours and days. The DSC sample was heated to 80 °C, cooled to −80 °C at 5.0 °C/min, and then heated to 80 °C at 5.0 °C/min under a nitrogen purge flow.

The WC of the polymers was calculated as follows:
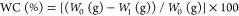
1where *W*_0_ and *W*_1_ are the weights of the hydrated and dried samples, respectively. *W*_1_ was determined after drying the post-DSC-measured samples in a vacuum oven at 150 °C. Because the latent heat of melting of water is 334 J/g, the amounts of intermediate water and non-freezing water (*W*_IM_ and *W*_NF_) were estimated from the following equations:
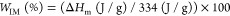
2
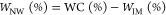
3where Δ*H*_m_ is the enthalpy change per gram of the hydrated samples during melting at less than 0 °C.^30^

### FTIR Analysis

The FTIR spectra were measured using the BL43IR beamline (SPring-8, Hyogo, Japan) with the approval of the Japan Synchrotron Radiation Research Institute (JASRI) (Proposal Number 2024A1128). The experimental procedure was the same as our previous report.^31^ Briefly, the dendrimer sample with 50% WC was sandwiched between two barium fluoride (BaF_2_) plates. The temperature of the sample was cooled from 80 to −80 °C and then heated from −80 to 80 °C at 10 °C/min rate. The spectra were obtained every 30 s during the cooling and heating processes.

### Cell Viability

HeLa cells (5 × 10^3^ cells/well) were cultured on a 96 well plate. After 1 day, SBM- and PSB-conjugated dendrimers (1 mg/mL) were added to each well. After the incubation for 24 h, cells were washed with PBS. A 3-(4,5-dimethylthiazol-2-yl)-2,5-diphenyl-2*H*-tetrazolium bromide (MTT) assay was performed to estimate the cell viability using MTT.^200^ The cell viability (%) was calculated from the percentage of the absorbance of cells treated with the dendrimers to that of intact cells. The same experiment was also examined using the dendrimer without SB compounds for a comparison.

### *In Vivo* and *Ex Vivo* Imaging

*In vivo* and *ex vivo* fluorescence imaging was performed as described in our previous report.^32^ First, the dendrimers were labeled with ICG, of which the bound number was estimated from ultraviolet–visible (UV–vis) measurements using the ICG molar extinction coefficient at 800 nm (ε = 147 000). 4T1 murine breast cancer cells (5 × 10^5^ cells) were subcutaneously injected into eight-week-old female BALB/c mice (Japan SLC, Inc., Shizuoka, Japan). After 2 weeks, the ICG-labeled dendrimers (18 nmol of ICG/kg in 100 μL of saline) were intravenously injected into the tumor-bearing mice. *In vivo* imaging was performed under anesthesia after 24 h using an IVIS Lumina Series III instrument (PerkinElmer, Inc., Waltham, MA, U.S.A.). Subsequently, the mice were euthanized, and tissues of interest, including the tumor, liver, and spleen tissues, were collected for *ex vivo* fluorescence imaging. For the second injection, the non-labeled zwitterionic dendrimers (0.1 mg/kg, 100 μL of saline) were intravenously pre-injected 7 day before the administration of the ICG-labeled dendrimers. Animal care, experiments, and euthanasia were approved by the Animal Care and Use Committee of Osaka Metropolitan University, and all animal procedures were performed in accordance with the committee guidelines.

## Results and Discussion

### Synthesis and Characterization of the SBM- and PSB-Conjugated Dendrimers

**Figure 2:**
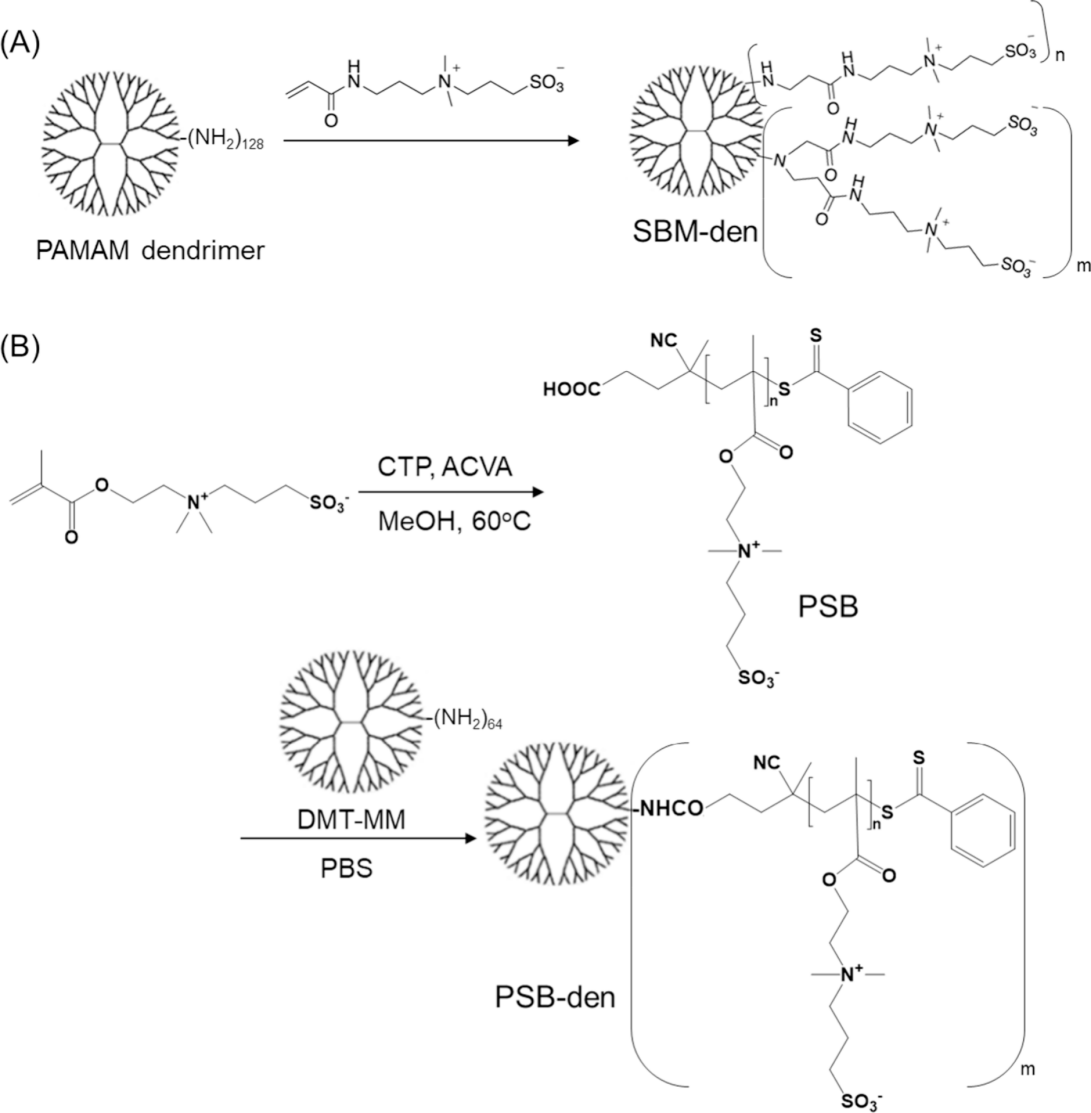
Synthetic schemes of (A) SBM-den and (B) PSB-den.

The synthetic procedures for SBM-den and PSM-den are shown in Figure 2. First, DMAAPS was used as a SBM, which reacted at 180 equiv with the PAMAM G5 dendrimer with 128 amino termini via Michael addition (Figure 2A). The synthesized SBM-den was characterized by ^1^H NMR spectroscopy. Each peak was assigned as shown in Figure S2 of the Supporting Information, and the bound number of SBM to the PAMAM dendrimer was estimated from the integral ratio of the SBM signals at 2.0 and 2.2 ppm to the dendrimer-derived signals (2–3 ppm) in the ^1^H NMR spectrum (Figure S2B of the Supporting Information). An equation was set up with the bound number of SBM as *n* based on the relation between the integral ratio and the proton number. When the equation was solved for *n*, the number of bound SBM was calculated as 178, which was almost the same as the amount of additive in the reaction mixture and larger than the number of terminal groups. This suggests that SBM reacted twice at some primary amino termini. For biodistribution analysis, labeling the dendrimer with a fluorescent probe is necessary. Because unreacted primary amino groups can be used for fluorescence dye labeling, the same reaction was performed by changing the SBM additive from 180 to 122 equiv. This was also characterized by ^1^H NMR spectroscopy (Figure S2C of the Supporting Information). The chemical shifts of some signals derived from the dendrimer and SBM changed, because the SBM reacted only once at the primary amino termini. The number of SBM molecules bound to the PAMAM dendrimer was estimated to be 107, in which several primary amino groups for labeling remained.

**Figure 3:**
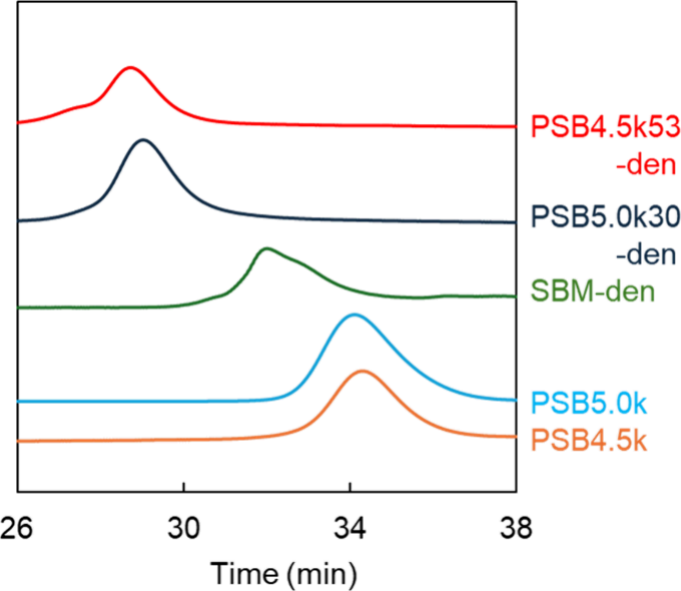
GPC chromatograms of PSB4.5k, PSB5.0k, and dendrimers conjugating SBM, PSB4.5k, and PSB5.0k.

Next, the PSB-dens were synthesized as follows (Figure 2B). Carboxyl-terminal PSB was synthesized via reversible addition–fragmentation chain transfer (RAFT) polymerization of DMAPS using CTP and ACVA as a RAFT agent and an initiator, respectively, according to previous reports.^33,34^ The same polymerization was carried out twice, and the synthesized PSBs were characterized by ^1^H NMR spectroscopy (Figure S3 of the Supporting Information). The degree of polymerization (DP) was estimated from the integral ratio of the SB signals at 4.5 ppm to the terminal CTP signal at 7–8 ppm. The DP of the obtained PSBs was calculated to be 15 and 17, which was similar to the monomer/RAFT agent ratio of 10. The molecular weights of the PSB with DP of 15 and 17 were calculated as 4500 and 5000, respectively, named as PSB4.5k and PSB5.0k. Then, the carboxy-terminal group of the PSBs was reacted with amino termini of the PAMAM dendrimer using a water-soluble condensation agent, DMT-MM, according to our previous report.^28^ We also attempted to characterize the synthesized PSB-dens using ^1^H NMR spectroscopy (Figure S4 of the Supporting Information). However, it was difficult to estimate the bound number because the PSB and dendrimer signals largely overlapped. Thus, a fluorescamine assay was performed to estimate the number of remaining primary amino groups, as described in our previous report.^28^ The number of bound PSB molecules was estimated by subtracting the remaining primary amino groups from the 64 termini. Our results indicate that 53 of PSB4.5k and 30 of PSB5.0k were conjugated to the PAMAM dendrimers. GPC was performed to examine the molecular weights of the synthesized dendrimers and polymers (Figure 3). This indicates that PSB-den was larger than PSB and SBM-den, confirming the synthesis of PSB-den. However, the molecular weights of the PSB estimated using the PEG standard were much smaller than those estimated from the NMR analysis. This may be because the ionic groups of PSB interact with the GPC column. Instead, the molecular weights of these dendrimers were calculated from the bound number and molecular weight of PSB (Table 1). These were larger than 30 kDa, which is a possible threshold of renal clearance.^35^

**Table 1:** Polymers Synthesized in This Study

compound	dendrimer (terminal number)	bound number of SB/PSB (in feed)	molecular weight (kDa)^a^	bound number of ICG^b^
PSB			4.5/5	
SBM-den	G5 (128)	178 (180 equiv)	78	
SBM-den-ICG	G5 (128)	107 (122 equiv)	59	0.6
PSB5.0k30-den(-ICG)	G4 (64)	30 (64 equiv)	166	(1.7)
PSB4.5k53-den(-ICG)	G4 (64)	53 (64 equiv)	252	(1.6)

a Calculated.

b The ICG-labeled dendrimers were used in animal experiments.

### Hydration Analysis of the SBM- and PSB-Conjugated Dendrimers

**Figure 4:**
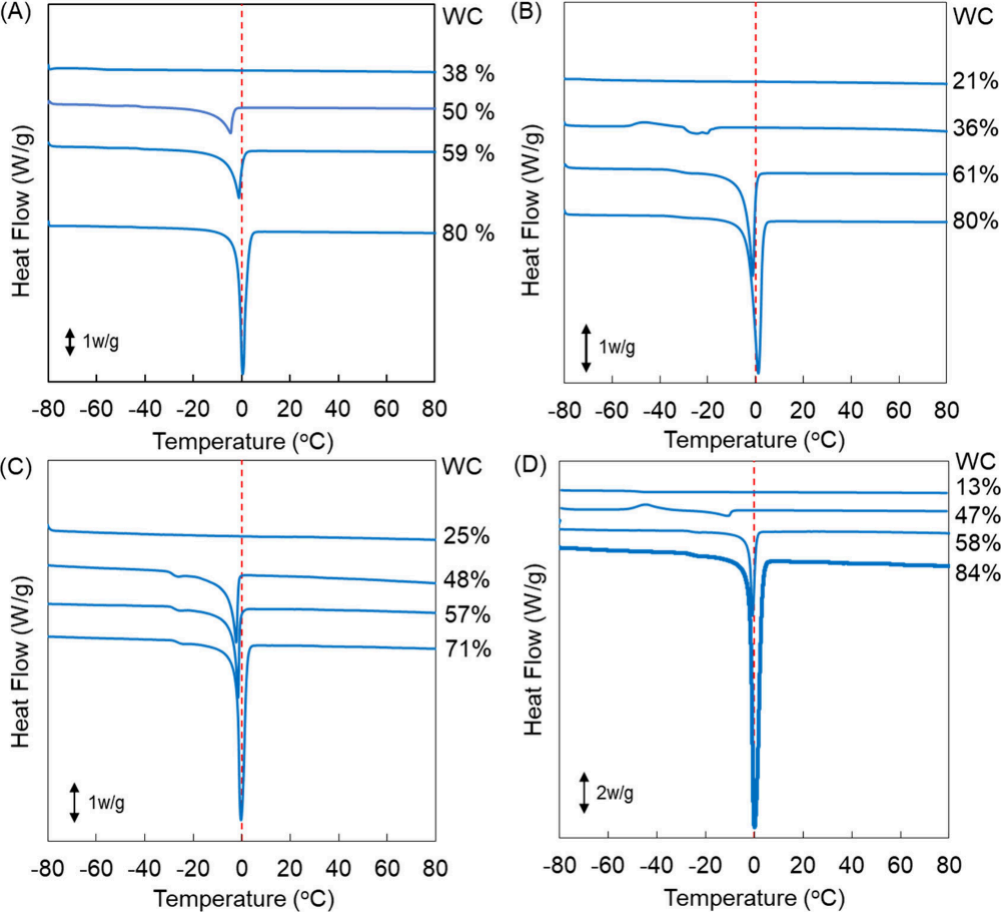
DSC curves of (A) SBM-den, (B) PSB, (C) PSB5.0k30-den, and (D) PSB4.5k53-den with different WCs during the heating process.

The hydration states of the SBM-den and PSB-dens were analyzed by DSC. SBM and PSB by themselves were also analyzed for comparison. The DSC thermograms of the compounds with different WC values during the cooling and heating processes are shown in Figures S5 and S6 of the Supporting Information and Figure 4. As shown in Figure 4, no obvious signals were observed at low WC, suggesting that all of the water molecules were non-freezing water. When the WC increased, a melting peak at less than 0 °C, corresponding to intermediate water, was observed. When the WC increased further, the melting peak enlarged and the peak top shifted to 0 °C, indicating the appearance of free water. The melting peak at less than 0 °C of SBM by itself was considerably small at 61% WC (Figure S5 of the Supporting Information). This indicates that the amount of intermediate water is poor and most water molecules are non-freezing water. Although the melting peak at less than 0 °C of SBM-den was still small, those of PSB and PSB-den were large (Figure 4). The maximum WC without free water was estimated as 50–60%. At around 55% WC, more than three independent DSC experiments were carried out to compare the average value of *W*_IM_ at the maximum WC in each sample (Table 2). *W*_IM_ of SBM-den was 20% at 54% WC. Our previous study showed that *W*_IM_ of the PAMAM dendrimer was 24% at 58% WC,^29^ indicating that modification with SBM did not increase *W*_IM_. In contrast, *W*_IM_ of PSB, PSB5.0k30-den, and PSB4.5k53-den were 26, 32, and 38% at 54–56% WC, respectively, indicating that PSB and PSB-den were rich in intermediate water. This also showed that modification of the dendrimer with PSB tended to increase *W*_IM_. This might be because the PSB molecules condensed on the surface of the dendrimer. The calculated densities were 0.5 and 0.8 chains/nm^2^ for PSB5.0k30-den and PSB4.5k53-den, respectively,^29,32^ which were compatible to or higher than the condensed polymer brush condition.^36^ The ratio of *W*_IM_/*W*_NF_ was also calculated. These indicate that SBM-den has fewer intermediate water than non-freezing water, but PSB-dens have more intermediate water than non-freezing water. The FTIR spectra of SBM- and PSB-conjugated dendrimers with 50% WC during the heating process were shown in Figure S7 of the Supporting Information. The FTIR spectra of SBM-den had the typical ice signals around 3260 cm^–1^ at less than −20 °C,^31^ whose peak decreased by the heating. On the other hand, those of PSB-den did not show the typical ice peak. This also suggests that the hydration behavior of PSB-den is different from that of SBM-den.

**Table 2:** *W*_IM_ and *W*_NF_ of SBM-den, PSB, and PSB-dens at Maximum WC without Any Free Water

sample	WC (%)	*W*_IM_ (%)	*W*_NF_ (%)	*W*_IM_/*W*_NF_
SBM-den	54 ± 3	20 ± 4	34 ± 1	0.59
PSB	55 ± 6	26 ± 7	29 ± 3	0.89
PSB5.0k30-den	54 ± 3	32 ± 2	22 ± 1	1.5
PSB4.5k53-den	56 ± 2	38 ± 1	18 ± 1	2.1

### Biodistribution of the SBM- and PSB-Conjugated Dendrimers

**Figure 5:**
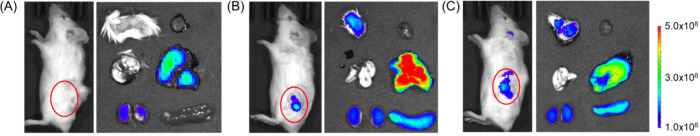
*In vivo* and *ex vivo* imaging of tumor-bearing mice intravenously injected with (A) SBM-den, (B) PSB4.5k53-den, and (C) PSB5.0k30-den. Circles in the *in vivo* images show the tumor. *Ex vivo* imaging of the tumor (left top), lung (left middle), kidneys (left bottom), heart (right top), liver (right middle), and spleen (right bottom) is shown. Rainbow bars show radiant efficacy.

Biocompatible nanoparticles, such as those modified with PEG and PEG alternatives, passively accumulate in tumor tissues via the EPR effect after intravenous injection.^3^ First, we examined the cytotoxicity of these SBM- and PSB-conjugated dendrimers compared to the PAMAM dendrimer without SB compounds (Figure S8 of the Supporting Information). SBM- and PSB-conjugated dendrimers showed essentially no cytotoxicity, confirming the biocompatibility. Next, the accumulation of zwitterionic dendrimers in tumors was examined using *in vivo* and *ex vivo* fluorescence imaging as described in our previous report.^32^ The ICG-labeled dendrimers were synthesized, and the *in vivo* and *ex vivo* imaging of tumor-bearing mice was performed at the 24 h post-injection of the dendrimers (Figure 5). Although SBM-den did not accumulate in the tumor, both PSB-dens accumulated in the tumor, suggesting that PSB-dens were retained in the blood and passively accumulated in the tumor via the EPR effect. *Ex vivo* imaging showed that SBM-den accumulated in the liver and kidney, but not in the tumor. PSB-den accumulated in the tumors, in addition to the liver and spleen. PSB-den, which was rich in intermediate water, accumulated in the tumor, whereas SBM-den, which was poor in intermediate water, did not accumulate in the tumor. Tumor accumulation was probably caused by the EPR effect involving blood circulation. Thus, our results suggest that the hydration state of zwitterionic dendrimers is related to their biodistribution. A similar relation was observed for PEGylated dendrimers,^29^ which supports our results for the zwitterionic dendrimers. Previous reports indicate that the intermediate water layer at the surface induces the repulsive force that works at the surface. Consequently, materials with rich intermediate water can suppress non-specific interactions in medical devices.^13−15^ It is possible that a carrier with a rich intermediate water shows prolonged blood circulation by a similar mechanism. Although our findings suggest that the hydration of zwitterionic dendrimers affects the biodistribution, some other factors, such as the molecular weight and surface charge, possibly affect the biodistribution. Comprehensive analysis using various dendrimers with different structures is required to elucidate the intermediate water concept in DDS materials.

**Figure 6:**
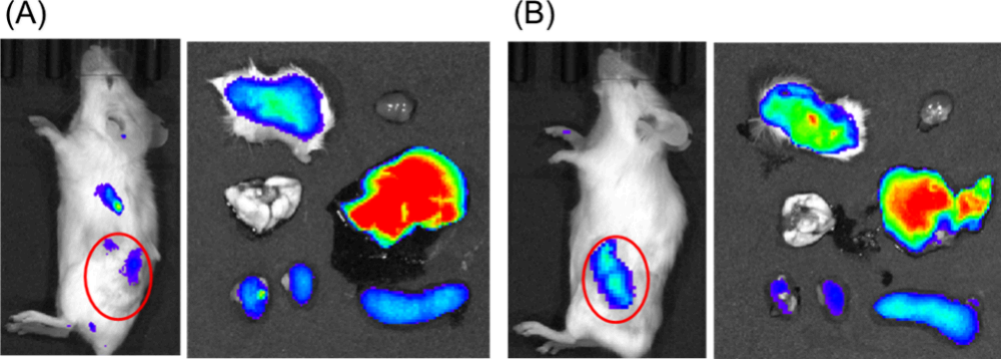
*In vivo* and *ex vivo* imaging of tumor-bearing mice intravenously injected with (A) PSB4.5k53-den and (B) PSB5.0k30-den after the second injection. Circles in the *in vivo* imaging, *ex vivo* imaging panels, and color scale are the same as Figure 5.

Finally, the ABC phenomenon of PSB-den was investigated. Pre-injection of non-labeled PSB-den was performed 7-day before the injection of ICG-labeled dendrimers into tumor-bearing mice. Figure 6 shows that PSB-den accumulated in the tumor after the second injection, suggesting that the ABC phenomenon was not observed. This suggests that PSB-den can overcome the ABC phenomenon, which is consistent with previous reports to indicate the biocompatibility of zwitterionic polymers.^3,7−10^ However, our results indicate that PSB-den accumulated more in the liver than in the tumor. Thus, improvement of zwitterionic dendrimers is necessary. The terminal structure of the polymer, the phenylcarbonothioylthio group, that came from the RAFT agent might decrease the biocompatibility of PSB-den. It is possible that the removal of the RAFT termini increases the tumor accumulation. Other types of zwitterionic polymers, such as MPC polymers and poly[2-[[2-(methacryloyloxy)ethyl]dimethylammonio]acetate], are known to be more biocompatible than poly(DMAPS),^7−10^ although we used a PSB, poly(DMAPS), as a model of zwitterionic polymers in this study. These polymers may be useful for the surface modification of dendrimers to improve tumor accumulation.

## Conclusion

We synthesized SBM- and PSB-conjugated dendrimers and analyzed their hydration states and biodistributions in tumor-bearing mice. The hydration states of the zwitterionic dendrimers conjugating the SB monomer and polymers differed significantly. PSB-den had more intermediate water than non-freezing water, whereas SBM-den had less intermediate water. Our animal experiments demonstrated that PSB-den but not SBM-den accumulated in tumors through the EPR effect. These results demonstrate a possible correlation between the hydration state and blood retention properties of the zwitterionic dendrimers. Our results suggest that the concept of intermediate water can be expanded to the design of drug carriers for DDSs. In addition, PSB-den accumulated in the tumor even after the second injection, suggesting that it might have overcome the ABC phenomenon. Because this property is indispensable for multiple administrations, our findings are useful for DDS in cancer treatment.

## Supporting Information Available

Standard curve in our fluorescamine assay (Figure S1), ^1^H NMR spectra of SBM-den (Figure S2), PSB (Figure S3), and PSB-den (Figure S4), DSC thermograms of SBM during the cooling and heating processes (Figure S5) and other zwitterionic compounds during the cooling process (Figure S6), FTIR spectra of the hydration samples of SBM-den and PSB-den (Figure S7), and cell viability of these dendrimers (Figure S8) (PDF)

## Abbreviations Used

DDS: drug delivery system
EPR: enhanced permeability and retention
PEG: poly(ethylene glycol)
ABC: accelerated blood clearance
MPC: 2-methacryloyloxyethyl phosphorylcholine
PB: phosphobetaine
SB: sulfobetaine
CB: carboxybetaine
DSC: differential scanning calorimetry
SBM: sulfobetaine monomer
PSB: polysulfobetaine (polySB)
FTIR: Fourier transform infrared spectroscopy
ICG: indocyanine green
DMAAPS: 3-[(3-acrylamidopropyl)dimethylammonio]propane-1-sulfonate
PAMAM: polyamidoamine
DMAPS: 3-[[2-(methacryloyloxy)ethyl]dimethylammonio]propane-1-sulfonate
CTP: 4-cyano-4-[(phenylcarbonothioyl)thio]pentanoic acid
DMT-MM: 4-(4,6-dimethoxy-1,3,5-triazin-2-yl)-4-methylmorpholinium chloride
PBS: phosphate-buffered saline
GPC: gel permeation chromatography
WC: water content
RAFT: reversible addition–fragmentation chain transfer
DP: degree of polymerization
MTT: 3-(4,5-dimethylthiazol-2-yl)-2,5-diphenyl-2*H*-tetrazolium bromide
*W*_IM_: amount of intermediate water
*W*_NF_: amount of non-freezing water
