# Supplementary material for: Hydration and Biodistribution of Zwitterionic Dendrimers Conjugating a Sulfobetaine Monomer and Polymers
Source: Langmuir. 2025 Jan 8;41(2):1411–7. doi: 10.1021/acs.langmuir.4c04276 (PMC11755784; doi:10.1021/acs.langmuir.4c04276)
Supplement: Supplementary file 1 — la4c04276_si_001.pdf [file la4c04276_si_001.pdf]

Supporting Information

Hydration and Biodistribution of Zwitterionic Dendrimers Conjugating Sulfobetaine Monomer and Polymers

Chie Kojima[1,2*], Rikuto Hirata[1], Nanako Dei[1], Hao He[1,2], Yuka Ikemoto[3], and Akikazu Matsumoto[1]

[1]Department of Applied Chemistry, Graduate School of Engineering, Osaka Metropolitan University, 1-1, Gakuen-cho, Naka-ku, Sakai, Osaka 599-8531, Japan

[2]Department of Materials Science and Engineering, School of Materials and Chemical Technology, Institute of Science Tokyo, 4259 Nagatsuta-cho, Midori-ku, Yokohama, Kanagawa 226-8503, Japan.

[3]Japan Synchrotron Radiation Research Institute, Spectroscopy Division, 1-1-1, Kouto, Sayo-cho, Sayo-gun, Hyogo 679-5198, Japan

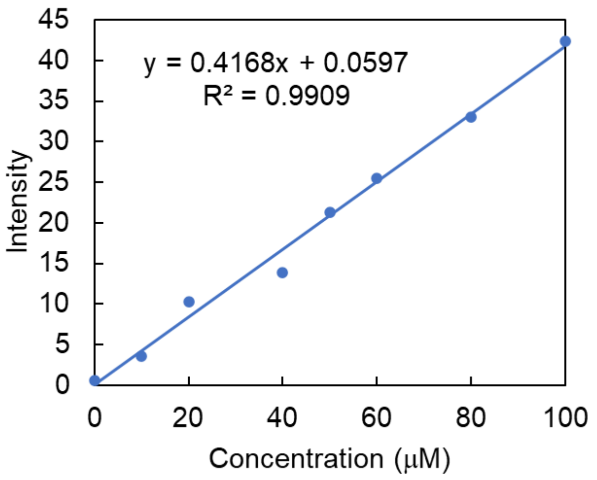

Figure S1. A typical standard curve in our fluorescamine assay.

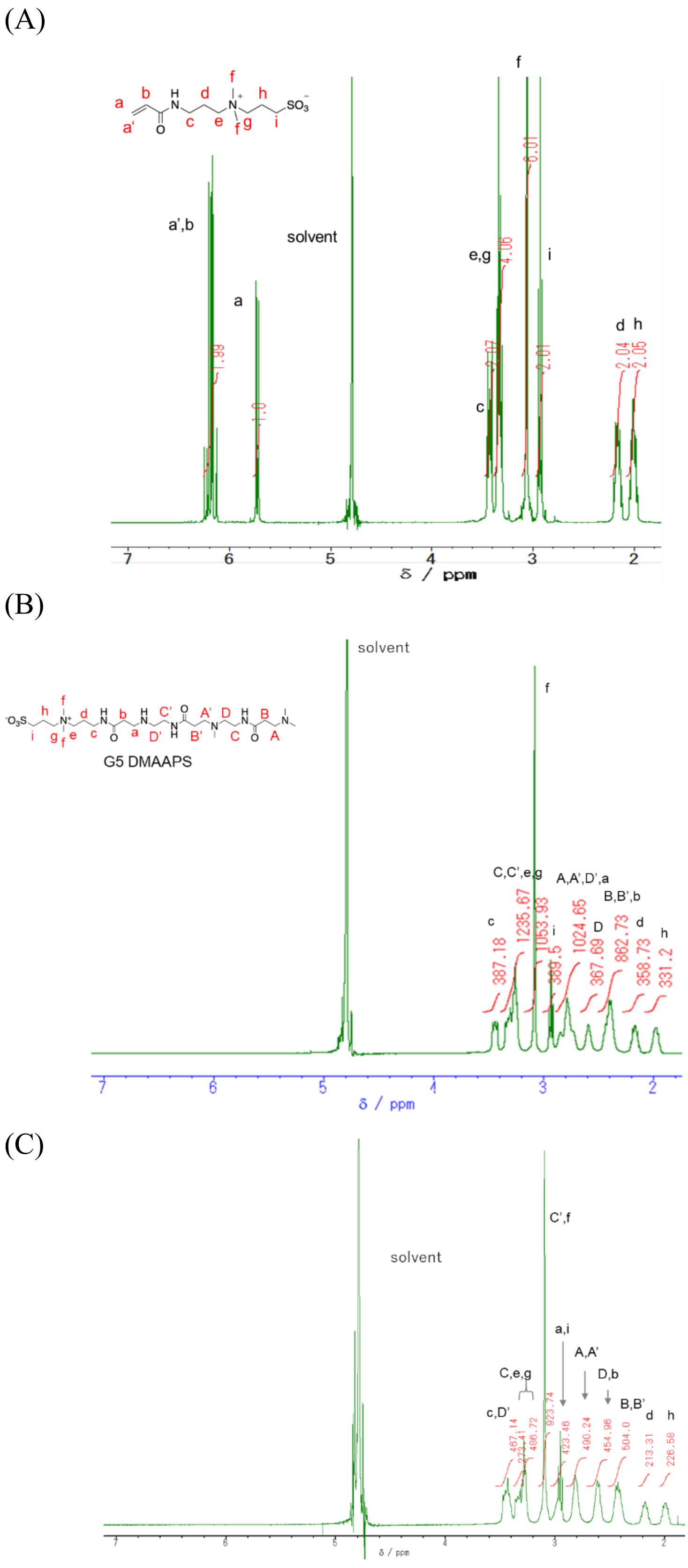

Figure S2. ^{1}H NMR spectra of DMAAPS (A) and SBM-dens reacted at 180 equiv. (B) and 122 equiv. (C) of DMAAPS in D_2O.

(A)

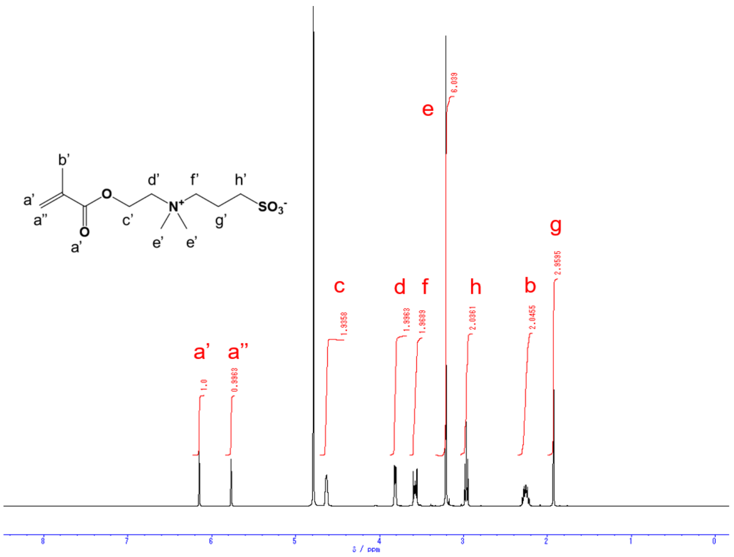

(B)

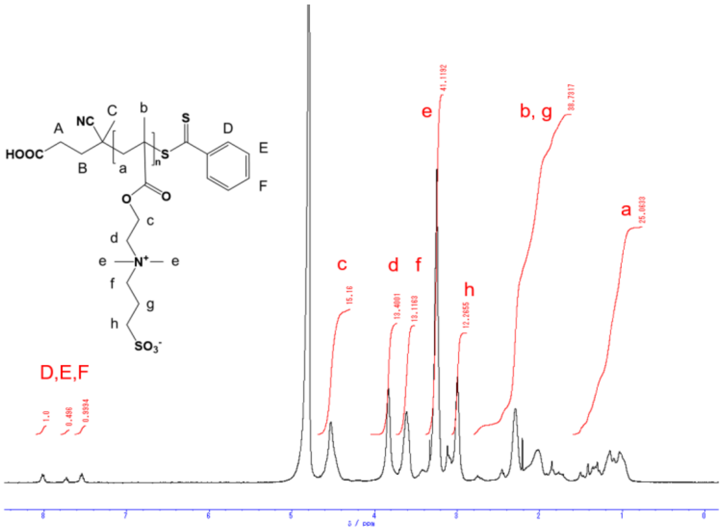

(C)

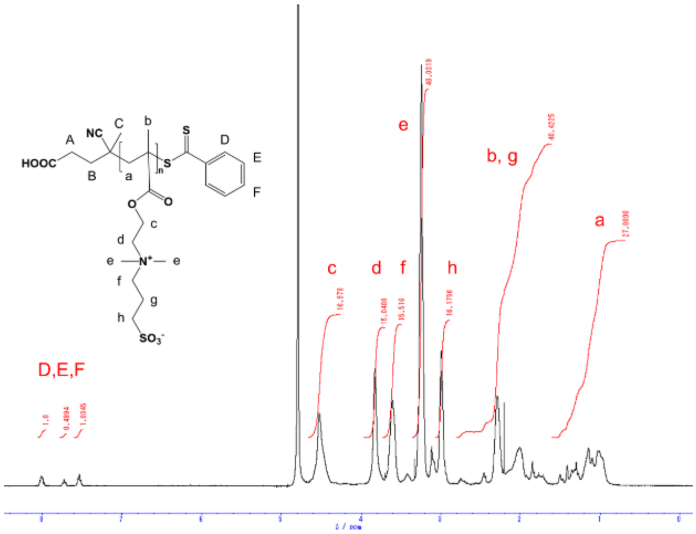

Figure S3. ^{1}H NMR spectra of DMAPS (A), PSB4.5k (B), and PSB5.0k (C) in D_2O.

(A)

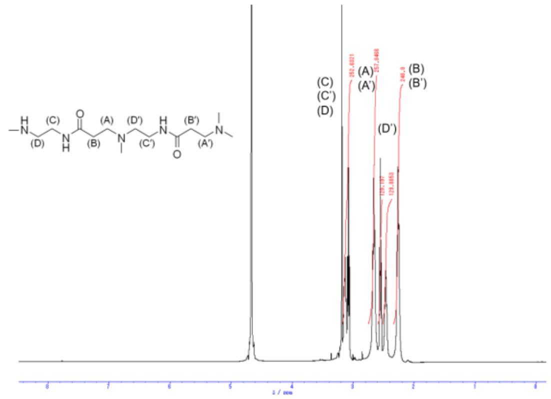

(B)

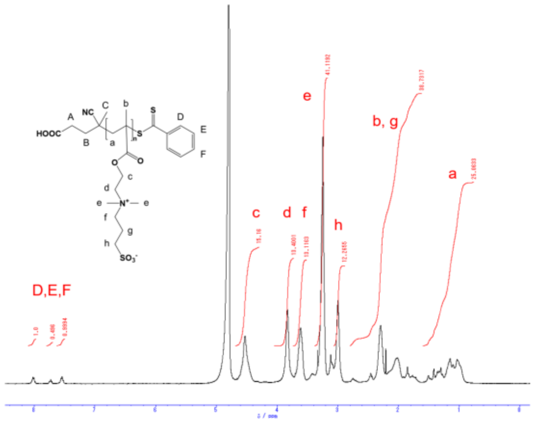

(C)

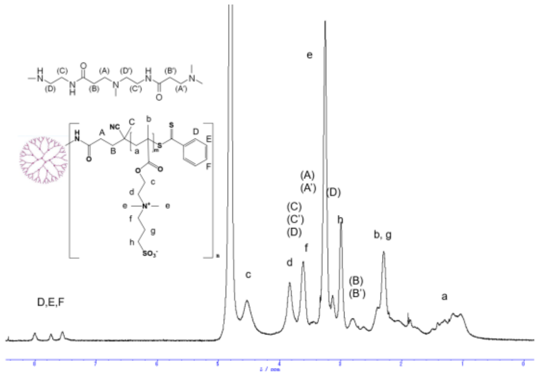

(D)

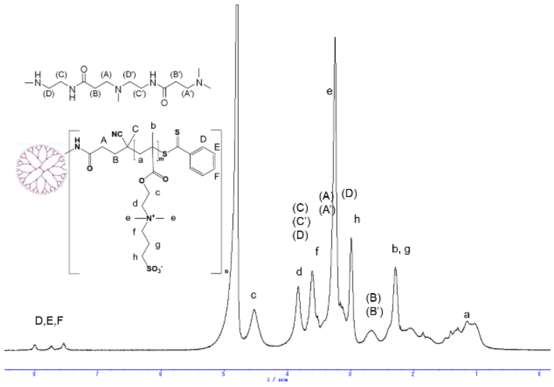

Figure S4. ^{1}H NMR spectra of PAMAM dendrimer only (A), PSB4.5k (B), PSB4.5k53-den (C), and PSB5.0k30-den (D) in D_2O.

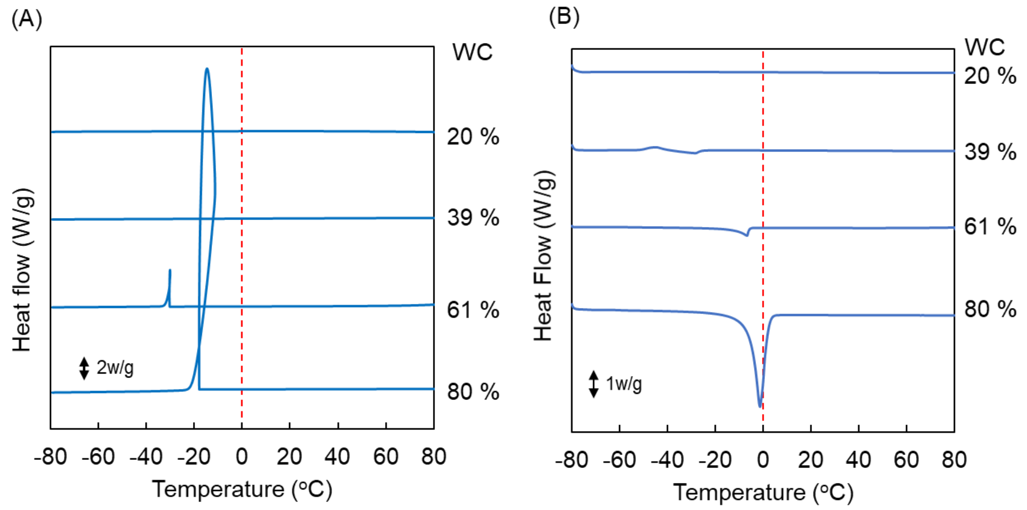

Figure S5. DSC curves of SBM with different water contents during the cooling (A) and heating (B) process.

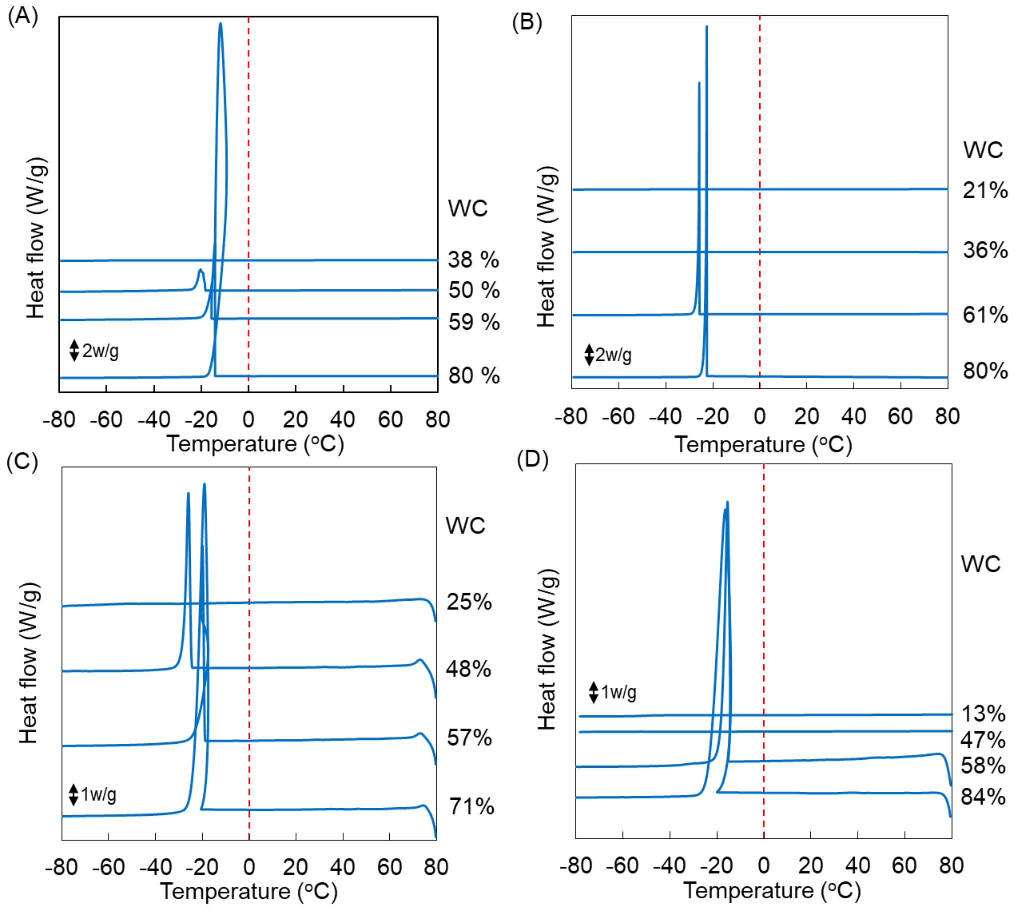

Figure S6. DSC curves of SBM-den (A), PSB (B), PSB5.0k30-den (C) and PSB4.5k53-den (D) with different water contents during the cooling process.

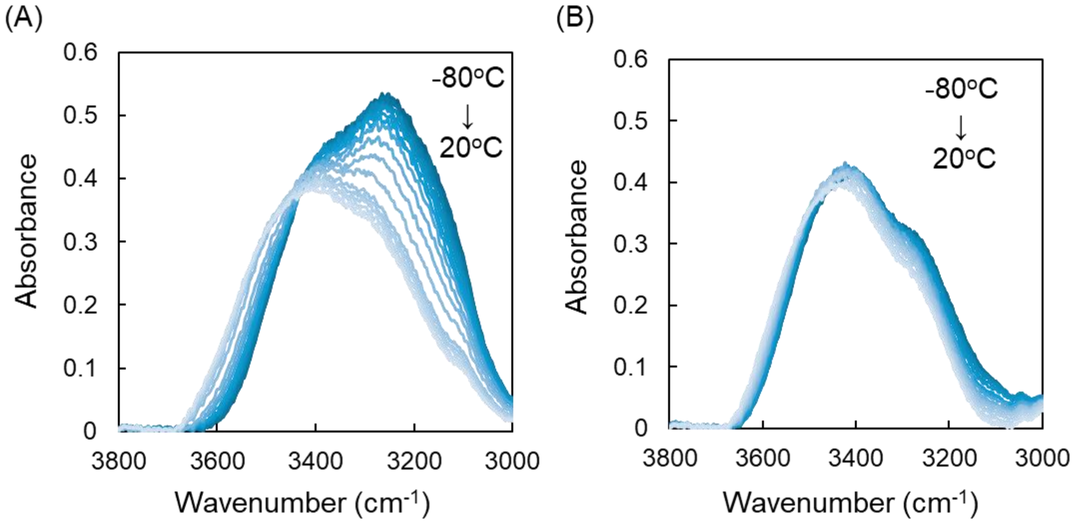

Figure S7. FT-IR spectra of (A) SBM- and (B) PSB-conjugated dendrimers with 50% WC during the heating process.

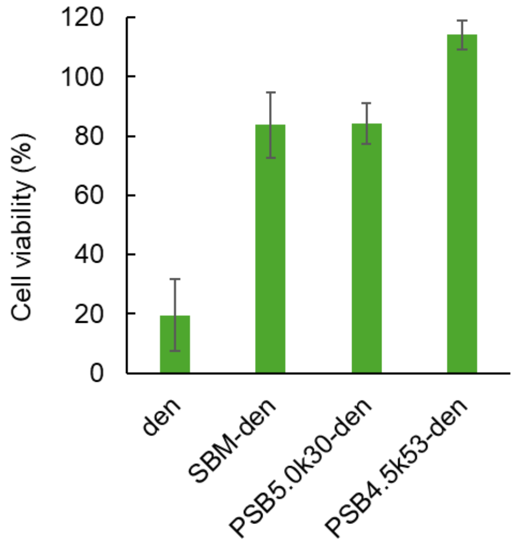

Figure S8. Cell viability of dendrimers with and without SB compounds (1 mg/mL).
